# Probing Charge-Controlled Inter-Domain Flexibility: Integrating Experimental and Coarse-Grained Approaches

**DOI:** 10.1021/acs.jcim.6c00989

**Published:** 2026-06-29

**Authors:** Larissa M. F. Adolfo, Rafael G. Viegas, Mario A. R. Pineda, Delaram Taghavi, Phelipe A. M. Vitale, Roberto K. Salinas, Vitor B. P. Leite

**Affiliations:** † São Paulo State University, São José do Rio Preto 15054-000, Brazil; ‡ Federal Institute of Education, Science and Technology of São Paulo (IFSP), Catanduva, São Paulo 15808-305, Brazil; § Bio-science School, Universidad Nacional de Colombia, Medellin 050034, Colombia; ∥ Institute of Chemistry, University of São Paulo, São Paulo 05508-220, Brazil; ⊥ Institute of Chemistry, São Paulo State University, Araraquara 14800-060, Brazil

## Abstract

The Na^+^/Ca^2+^ exchanger (NCX) is a membrane protein that couples the downhill transport of Na^+^ across the plasma membrane to the uphill movement of Ca^2+^ in the opposite direction. The NCX is a key Ca^2+^ extrusion mechanism in excitable cells. It contains a transmembrane domain that catalyzes the counter-transport of Na^+^ and Ca^2+^, and a large intracellular loop (IL) that is responsible for the allosteric regulation of the exchanger by its substrates. The NCX intracellular loop contains a two-domain Ca^2+^-sensor, CBD12, which harbors Ca^2+^ regulatory sites. Ca^2+^-binding to CBD12 triggers NCX activation and alleviates Na^+^-dependent inactivation. An outstanding question in this field is how Ca^2+^-binding to CBD12 activates the exchanger? Previous experimental studies showed that CBD12 displays considerable interdomain flexibility in the unbound state, while Ca^2+^ binding near the linker between the two domains stabilizes a rigid and widely opened interdomain conformation. This phenomenon could be an important step in the Ca^2+^ regulation mechanism. Using the Drosophila exchanger, CALX, as a model system, we carried out coarse-grained molecular dynamics simulations using a dual-basin structure-based model (SBM) to sample large-scale conformational transitions between open and closed states. In addition, we calculated the CBD12 free energy profile along the open-closed transition coordinate, in the Ca^2+^-bound and in the free states. We found that Ca^2+^ binding reshapes the CBD12 free energy landscape, stabilizing a widely opened interdomain conformation in agreement with previously published experimental data. Notably, the energy landscape of the two CALX CBD12 isoforms, which differ by only five amino acids near the interdomain linker, is substantially different. These results provide atomistic insights into the open-closed conformational transition experienced by this two-domain construct, and are consistent with the considerable broadening of the solution NMR resonances of CBD12 1.2 in comparison with the 1.1 isoform.

## Graphical abstract

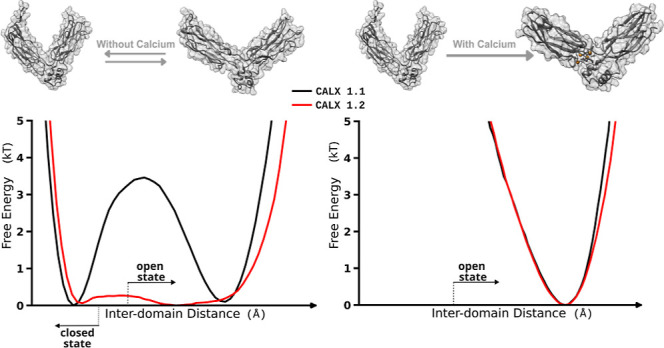

## Introduction

The Na^+^/Ca^2+^ exchanger (NCX) is a secondary active transporter that couples the downhill transport of Na^+^ across the plasma membrane with the uphill movement of Ca^2+^ in the opposite direction.^1,2^ The NCX is an important Ca^2+^ extrusion mechanism, especially in excitable cells. The *Drosophila* Na^+^/Ca^2+^ exchanger, CALX, is the primary Ca^2+^ extrusion mechanism in sensory olfactory neurons,^3,4^ and plays a key role in the various steps of the phototransduction cascade in photoreceptor cells.^5^

NCX and CALX exchangers contain a transmembrane domain, which transports Na^+^ and Ca^2+^ through the lipid bilayer, and a large intracellular loop that is responsible for the allosteric regulation of the exchangers by their substrates, Na^+^ and Ca^2+^.^1,6,7^ The large intracellular loop contains two adjacent Ca^2+^-binding domains, CBD1 and CBD2, which harbor regulatory Ca^2+^ binding sites.^8,9^ The two CBDs share a Calxβ motif,^3,10^ which is formed by seven β-strands arranged in two β-sheets (G′-A-B-E and C-D-F-G-A′) forming a β-sandwich.^11–13^ The Ca^2+^-binding sites are located in the distal interstrand loops of the β-sandwich.^13^ CBD1 is the primary Ca^2+^-binding domain of the Na^+^/Ca^2+^ exchangers. It binds four Ca^2+^ ions with dissociation constants *K*_d_ ≈ 0.3–2.3 μM and high cooperativity (*n*_Hill_ ≈ 2.1–2.9).^13–16^ CBD2 is the secondary Ca^2+^-binding domain. Its Ca^2+^-binding affinity and stoichiometry vary according to the NCX gene (NCX1–NCX4) and the splicing variant isoform. The two CBDs are connected by a short three-amino acid linker, forming a two-domain Ca^2+^-sensor called CBD12.^17^ Ca^2+^-binding to CBD12 triggers NCX activation^14,18–20^ but inhibits CALX.^11,21,22^ The *CALX* gene displays two alternative splicing variants, *CALX* isoforms 1.1 and 1.2, which differ in a short segment of five amino acids in the CBD2 FG-loop.^23^ Although the two CALX isoforms exhibit negative Ca^2+^ regulation, steady-state and peak currents are allosterically inhibited to a greater extent in the 1.1 than in the 1.2 isoform.^24^

Crystallographic structures showed that the CBD1 Ca^2+^ regulatory sites are located near the linker with CBD2 (Figure 1).^8,9^ NMR spectroscopy and SAXS data showed that CALX isoform 1.1 CBD12 and the NCX1.4 CBD12 exhibit considerable interdomain flexibility in the unbound state.^25–27^ In contrast, Ca^2+^ binding to CBD1 stabilizes an extended interdomain arrangement between the two CBDs as seen in crystallographic structures. This dynamic behavior seems to be preserved in the full-length exchanger. Hydrogen–deuterium exchange mass spectrometry (HDX-MS) experiments carried out with NCX1.4 showed that Ca^2+^-binding significantly restricts the dynamics of the CBD12 sensor domain in the full-length protein.^28^

**Figure 1:**
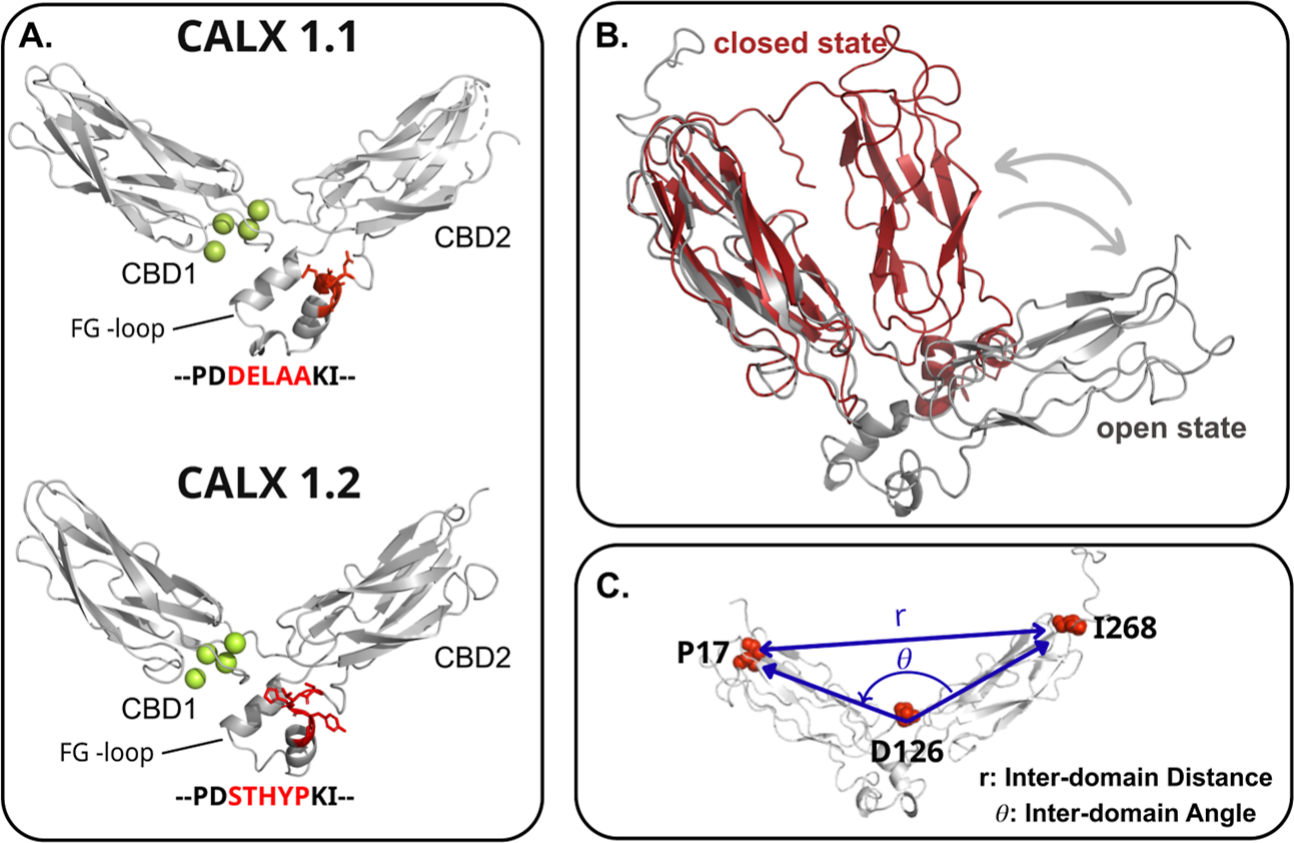
(A). Three-dimensional representation of CBD12 from CALX isoforms 1.1 (PDB ID: 3RB5) and 1.2 (PDB ID: 3RB7) in the Ca^2+^-bound state.^8,9^ The regions shown in red highlight the sequence and structural differences between the isoforms, while the green spheres indicate the four calcium ions resolved in the X-ray crystal structure. (B). Representation of the interdomain motions that occur in the absence of calcium, with the open state depicted in gray and the closed state in burgundy. (C). Definition of the structural parameters analyzed: *r* (interdomain distance, Å) and θ (interdomain angle, degrees). In the gene numbering scheme P17 is P441, D126 is D550 and I268 is I692.

Recent cryoelectron microscopy structures of the full-length human cardiac NCX1 in complex with a Fab antibody fragment revealed the overall architecture of the exchanger.^29,30^ CBD12, which makes most part of the large intracellular loop, is seen in the wide open conformation with CBD2 positioned close to the transmembrane domain, making interactions with a four-stranded β-sheet (β-hub) near the membrane interface, effectively locking the exchanger in an inactive state. Ca^2+^ binding to CBD2 disrupts these interactions, releasing CBD12 from this position and leading to activation. However, because in these structures the CBD1 Ca^2+^-binding sites were fully occupied, they do not explain how Ca^2+^-binding to CBD1 leads to NCX allosteric activation and CALX inactivation.^28^ Moreover, CALX-CBD2 does not bind Ca^2+^ at physiological concentrations,^12^ hence the only Ca^2+^ regulatory sites in this exchanger are located in the CBD1 domain.

Conventional all-atom molecular dynamics (MD) simulations performed by Souza-Degenhardt and co-workers (2021) successfully characterized the structural features of the open and closed states of CALX CBD12.^27^ However, spontaneous transitions between these states were not observed, likely due to intrinsic time scale limitations. To overcome these limitations, we employed a dual-basin structure-based model (SBM). This approach enabled the exploration of large-scale conformational transitions between open and closed states and the computation of the CBD12 free energy profile, both in the presence and absence of Ca^2+^ and for the CALX 1.1 and 1.2 isoforms. The energy landscape visualization method (ELViM) analysis was performed to project the conformational ensembles onto a reduced space, enabling a clear visualization of the conformational landscape and the transition between open and closed states. This approach allowed for the identification of well-defined conformational regions and the selection of representative structures associated with distinct basins. Our results provide a mechanistic description of the open–closed transition that was not accessible in previous atomistic simulations, revealing how differences in the underlying energy landscape modulate the conformational dynamics of the two CBD12 isoforms. Altogether, these results are consistent with the view that these two CBD12 isoforms exhibit distinct intrinsic flexibility in the absence of Ca^2+^ as suggested by experimental solution NMR data.

## Materials and Methods

### Cloning, Expression and Purification

A DNA fragment corresponding to the CALX1.1 (UniProt A0A0B4K6R7) CBD12 domain (residues 434 to 697) was cloned into the pAE vector for protein expression as a fusion with a six-histidine N-terminal tag and two extra amino acids from the vector (MHHHHHHLE) as described previously.^27,31^ The CBD12 1.2 isoform was cloned by mutating amino acids 651–655 of this clone to STHYP by PCR overlapping. The constructs CBD12 1.1 and 1.2 were expressed in *Escherichia coli* strains C43­(DE3) (CBD12 1.2) or BL21­(DE3) RIL (CBD12 1.1), in M9 minimal medium supplemented with 0.5 g/L of ^15^NH_4_Cl (Cambridge Isotopes Laboratories, CIL), and purified as described.^27^ The protein concentration in the NMR samples was calculated from the absorbance at 280 nm assuming ε = 21890 M^–1^ cm^–1^ (CBD12 1.2) or 20400 M^–1^ cm^–1^ (CBD12 1.1).

### NMR Spectroscopy

^1^H–^15^N BEST-TROSY NMR spectra were acquired on a Bruker AVANCE III spectrometer equipped with a TCI cryoprobe and operating at 800 MHz (^1^H Larmor frequency). The two spectra were recorded with 1024 × 128 complex points and extended to 2048 × 512 by zero-filling. All dimensions were multiplied by a cosine square apodization function. NMR spectra were processed with TopSpin (Bruker Biospin) and analyzed using CCPNMR-Analysis.^32^ NMR samples contained approximately 96 and 80 μM of CBD12 1.1 or 1.2, in 20 mM Tris–HCl pH 7.5, 1 mM of β-mercaptoethanol, 1 mM of PMSF, 2 mM of EDTA, 200 mM of NaCl, 1% of glycerol, and 10% of D_2_O.

### Molecular Dynamics Simulations

All simulations were performed using an all-atom structure-based model (SBM)^33^ representing heavy atoms as unit-mass beads. Separate models were constructed for isoforms CBD12–1.1 and CBD12–1.2. Bond lengths, bond angles, improper dihedrals, and planar dihedrals were maintained using harmonic potentials, with equilibrium values and force constants defined according to the SMOG parametrization.^34^ Native contacts were defined using the Shadow map algorithm, which accounts for occlusion effects. A probe radius of 6 Å was used, and contacts were assigned based on unobstructed line-of-sight between heavy atoms.^35^

To explore the conformational landscape connecting the open and closed states, we employed a dual-basin Go̅-like model,^36^ in which the potential energy surface contains two minima corresponding to the reference open and closed conformations. The reference structures defining these states were extracted from the molecular dynamics simulations reported by de Souza Degenhardt et al.,^27^ where conformational ensembles for CBD12–1.1 were refined and validated against SAXS and ^1^H–^15^N residual dipolar coupling (RDC) measurements. Briefly, MD-generated conformers were filtered according to their agreement with RDC data and subsequently used in EOM analysis to identify subensembles consistent with the SAXS profiles. Thus, the closed reference structure for CBD12–1.1 corresponds to an experimentally supported interdomain arrangement representative of the dominant states sampled in solution. In contrast, the closed reference conformation for CBD12–1.2 was obtained from MD simulations, as described in the Supporting Information (see Figure S4). The transition between these conformational states was characterized based on two collective variables: the interdomain distance, defined as the Cα–Cα distance between residues P17 (P441) and I268 (I692), and the interdomain angle defined by the Cα atoms of residues P17 (P441), D126 (D550), and I268 (I692) (Figure 1C). Distances of approximately 80 Å (∼120^°^) characterize the open conformation, whereas distances near 20 Å (∼20^°^) correspond to the closed conformation.

For a native contact between the atoms *i* and *j*, interactions present in only one of the two conformational states (open or closed) were modeled using a single-Gaussian potential. The interaction energy is defined as

1 $$V_{ij}^{single}\left(r_{ij}\right)=\epsilon_{ij}^{open/closed}\left(\left(1+\frac{r_{NC}^{12}}{\epsilon_{ij}^{open/closed}r_{ij}^{12}}\right)\left[1+G\left(r_{ij},r_{ij}^{0}\right)\right]-1\right)$$

where

2 $$G\left(r,r^{0}\right)=-\exp\left[-\frac{{\left(r_{ij}-r_{ij}^{0}\right)}^{2}}{2\sigma_{ij}^{2}}\right]$$

where, *r*_*ij*_ is the instantaneous distance between atoms *i* and *j*, *r*_*ij*_^0^ is the native contact distance in the reference structure where the contact is present, corresponding to either the open or the closed state. The parameter ϵ_*ij*_ defines the depth of the attractive well associated with that state (ϵ_*ij*_^open^ or ϵ_*ij*_^closed^). The *r*_NC_ defines the excluded-volume contribution associated with non-native interactions, whereas σ_*ij*_ controls the width of the Gaussian well.

For contacts shared by both conformational states, interactions were described using double-Gaussian potentials defined as

3 $$V_{ij}\left(r_{ij}\right)=\epsilon_{ij}\left(\left(1+\frac{r_{NC}^{12}}{\epsilon_{ij}r_{ij}^{12}}\right)\left[1+G\left(r_{ij},r_{ij}^{open}\right)\right]\left[1+G\left(r_{ij},r_{ij}^{closed}\right)\right]-1\right)$$

The equilibrium distances *r*_*ij*_^open^ and *r*_*ij*_^closed^ correspond to the native contact distances extracted from the respective reference structures. The Gaussian widths σ_*ij*_ were assigned according to the default dual-basin parametrization implemented in SMOG2,^34^ and the parameter ϵ_*ij*_ defines the depth of the attractive well for shared contacts.

### Simulation Protocol

Simulations were performed using the SMOG 2.4.5 package^37^ via the OpenSMOG interface^38^ using a Langevin integrator (time step 0.002). The folding temperature (*T*_*f*_) of the system was first estimated by conducting simulations of the standard single-basin SBM at 10 temperatures spanning the unfolded to the folded state. *T*_*f*_ was identified by the peak of the heat capacity (*C*_v_(*T*)), calculated through the weighted histogram analysis method (WHAM).^39^ All subsequent thermodynamic and conformational properties were then characterized using dual-basin SBM. For production runs, five independent replicas were performed for each system at a temperature of 0.8*T*_*f*_, with 2 × 10^8^ simulation step for each replica. Trajectories, total energies, and individual energy components were recorded every 10^3^ steps, yielding approximately 2 × 10^5^ frames per replica (corresponding to approximately 2 μs).

To ensure comparable sampling of the open and closed states, the interaction strengths (ϵ_*ij*_) defining the depth of the contact potentials in each basin were systematically adjusted. In general, the resulting free-energy profiles are sensitive to these parameters, often favoring a single dominant basin. Therefore, for each isoform, ϵ_*ij*_^open^ and ϵ_*ij*_^closed^ were independently calibrated to yield nearly degenerate free-energy minima for the open and closed states, ensuring balanced sampling of both conformations and adequate exploration of the transition region.

This procedure was adopted to enable a consistent comparison of the effects of Ca^2+^ binding on stability and flexibility, minimizing biases arising from intrinsically unbalanced baseline landscapes. While this calibration enforces comparable thermodynamic stability between states, the free-energy barriers between them differ between isoforms due to intrinsic features of the model. For isoform 1.1, ϵ_*ij*_^closed^ and ϵ_*ij*_^open^ were set to 0.7 and 1.3, respectively, whereas for isoform 1.2 the corresponding values were 0.65 and 1.35. For both isoforms, the attractive well depths of shared contacts were fixed at ϵ_*ij*_ = 1.

### Dual-Basin SBM with Debye–Hückel (DH) Interactions and Ion Coordination

We performed additional simulations including electrostatic interactions using a Debye–Hückel potential to implicitly account for ionic screening.^40^ Ionizable residues were assigned integer charges corresponding to their dominant protonation states at physiological pH (7.4).^41^ Aspartate and glutamate were modeled as deprotonated (−1), whereas lysine and arginine were modeled as protonated (+1). Electrostatic interactions between charged sites were described by

4 $$V_{DH}\left(r_{ij}\right)=k_{ele}\frac{q_{i}q_{j}}{\varepsilon_{r}r_{ij}}\exp\left(-\kappa r_{ij}\right)$$

where *q*_*i*_ and *q*_*j*_ are the charges of sites *i* and *j*, *r*_*ij*_ is their distance, *k*_ele_ = 322 kcal·Å·mol^–1^·e^–2^ is the Coulomb constant, ε_*r*_ = 80 is the relative dielectric constant,^40,42^ and κ is the inverse Debye length. For monovalent salt concentrations (*C*_*s*_), the inverse of the Debye length is given by $\kappa \approx 3.2\sqrt{C_{s}}\cdot {nm}^{-1}$ .^42–44^ Considering a concentration of 100 mM, at room temperature and physiological pH, κ ≈ 1.0 nm^–1^. To improve computational efficiency, the Debye–Hückel interactions were truncated at a cutoff distance of *r*_c_ = 2.0 nm, which is approximately twice the Debye screening length under these conditions, ensuring that the neglected contributions are negligible.

As the introduction of electrostatics alters the energy balance of the system, the attractive well depths (ϵ_*ij*_^open^ and ϵ_*ij*_^closed^) were adjusted to maintain comparable probabilities for the open and closed states. For isoform 1.1, simulations were performed at 0.8*T*_*f*_ with the attractive well depths ϵ_*ij*_^closed^ and ϵ_*ij*_^open^ set to 0.73 and 1.3 respectively. In contrast, for isoform 1.2, simulation temperature was set to 0.9*T*_*f*_, with ϵ_*ij*_^closed^ and ϵ_*ij*_^open^ set to 0.65 and 1.3, respectively.

### Inclusion of Calcium Ions

Calcium ions (Ca^2+^) observed in the experimental crystal structures were explicitly included in the dual-basin SBM within Debye–Hückel interactions. For isoforms 1.1 and 1.2, the crystal structures with PDB IDs 3RB5 and 3RB7, respectively, were used to define the number of bound calcium ions and their spatial arrangement relative to the protein. The coordinating residues were identified directly from the experimental structures, ensuring that calcium binding sites were defined based on native-state geometry. The interaction between each Ca^2+^ ion and its coordinating residues was modeled using harmonic distance restraints.

To represent the fully calcium-bound state, harmonic distance restraints were introduced between each Ca^2+^ ion and its coordinating atoms. Interactions were included for ion–atom pairs separated by less than 6.0 Å in the reference structure. The potential energy associated with each ion–atom interaction was defined as

5 $$V_{ion}\left(r\right)=\frac{1}{2}k{\left(r-r_{0}\right)}^{2}$$

where *r* is the instantaneous distance between the calcium ion and a coordinating atom, *r*_0_ is the equilibrium distance extracted from the experimental structure, and *k* = 1000 kcal mol^–1^ nm^–2^ is the force constant controlling the stiffness of the restraint. This formulation stabilizes the native calcium coordination geometry while still allowing thermal fluctuations around the experimental binding configuration. The parametrization followed established approaches used in structure-based models incorporating explicit divalent ions.^45,46^

### Free Energy Calculation

For each trajectory frame, the interdomain distance (*r*) and interdomain angle (θ) were measured. The corresponding free-energy profiles were then obtained from the probability distributions *P*(*r*) or *P*(θ) via Boltzmann inversion

6 $$F\left(x\right)=-k_{B}T\;\ln P\left(x\right)$$

The free-energy profiles were computed independently for each replica using a consistent binning scheme and probability density normalization. The reported profiles correspond to the mean over five independent simulations, and uncertainty was estimated as the standard deviation across replicas. Shaded regions representing ±2 standard deviations are shown to quantify statistical variability. Convergence of the free-energy profiles was assessed by comparing profiles obtained from increasing fractions of the trajectories.

### Energy Landscape Visualization Method

ELViM is a methodology for describing the energy landscape of biomolecules without relying on a predefined reaction coordinate. The approach involves: (i) the definition of a structural dissimilarity metric; (ii) the construction of the associated dissimilarity matrix; (iii) the reduction of the N-dimensional space into a two-dimensional projection; and (iv) the analysis of the resulting projection using different approaches.^47,48^

Each dot in the ELViM projection represents a single conformation, and the distance between dots optimally reflects the estimated structural dissimilarity between conformations. The density of states was estimated from the ELViM projection using Gaussian kernel density estimation (KDE), as implemented in SciPy,^49^ with the bandwidth adjusted to avoid over- or under-smoothing. High-density regions correspond to the most populated conformational states and were represented by local conformational signatures (LCS), defined as centroid conformations that minimize the average distance-rmsd (dRMSD) within each region. Further details of this procedure can be found in ref 50.

## Results and Discussions

For electrostatic-free and calcium-free simulations, we employed an all-atom structure-based model in which the contact potential was defined using dual-basin potentials to allow the protein to transition between the open and closed states. As described in the Methods section, the parameters ϵ_*ij*_^open^ and ϵ_*ij*_^closed^ were calibrated in the absence of ions to ensure comparable sampling of the open and closed basins for both isoforms. The free-energy profiles along the interdomain angle (Figure 2A) reveal a marked difference in the open/closed transition barrier between the two isoforms. The energy barrier is approximately 3 *k*T higher in isoform 1.1 (black curve) than isoform 1.2 (red curve). The lower barrier in isoform 1.2 indicates that transitions between the two states are energetically more accessible in this isoform. Although coarse-grained structure-based models are not expected to provide quantitatively exact free-energy barriers, relative changes in the landscape can still be physically meaningful for large-scale collective motions.^51^ In particular, transition times depend exponentially on the barrier height, τ ∝ exp­(−Δ*G* ^‡^/*k*_*B*_ *T*), such that a difference of ∼3*k*_B_ *T* may correspond to an approximately 20-fold change in the characteristic transition time scale. Similar effects have recently been observed in structure-based simulations of ribosomal dynamics.^52^ Consequently, isoform 1.2 can sample a wider range of interdomain angles. Consistent free-energy profiles were obtained for increasing trajectory lengths, indicating satisfactory convergence of the conformational landscape (Supplementary Figure S2). This increased mobility becomes evident when comparing Figure 2C with Figure 2B, where we present the density plot of the radius of gyration (*R*_*g*_) as a function of interdomain angles. The heterogeneity of states for isoform 1.2 is noticeably broader when looking at the *R*_*g*_ axis.

**Figure 2:**
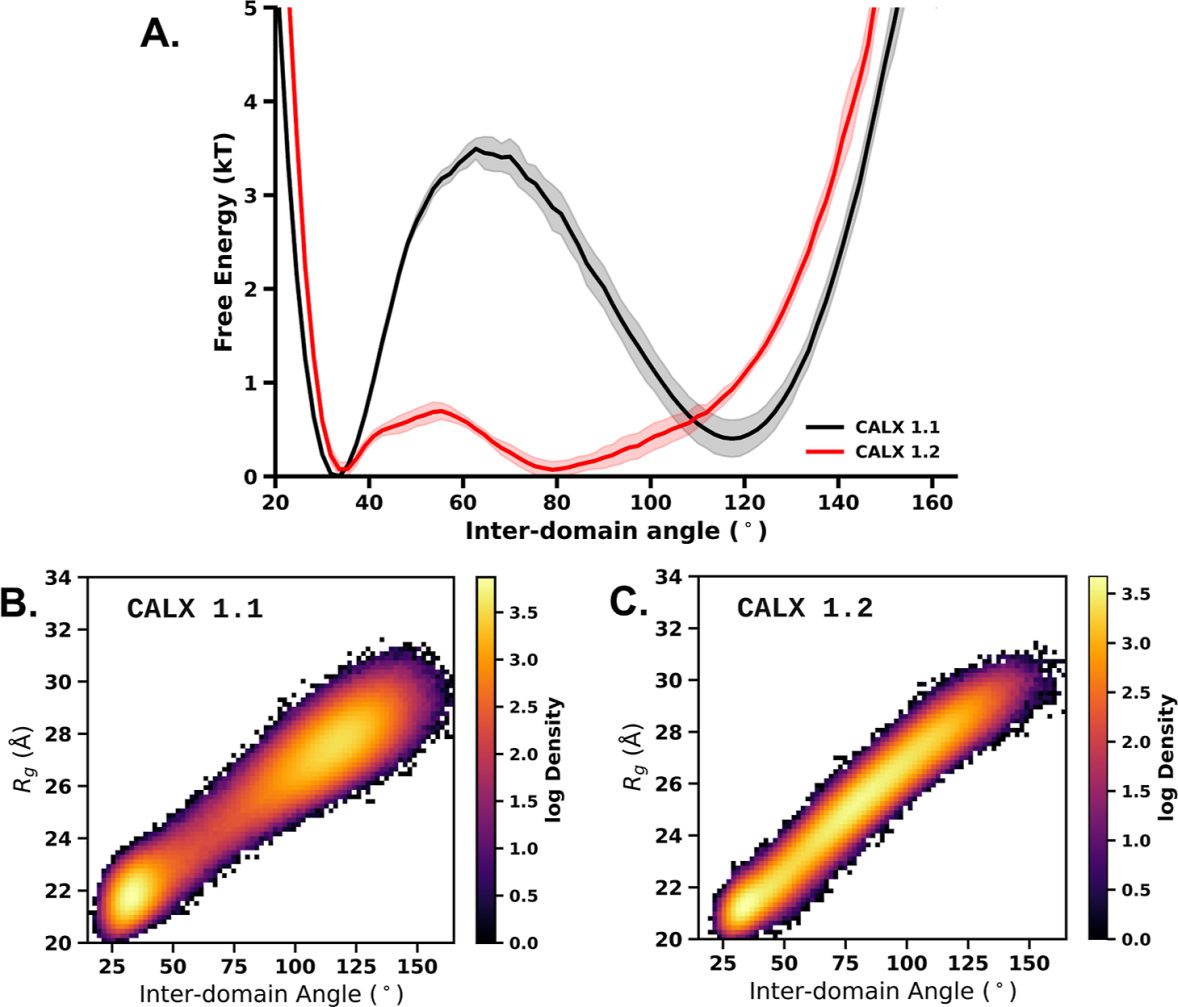
All-atom dual basin SBM without electrostatics (no charges, no ions). (A). Free-energy profiles as a function of the interdomain angle. The black and red curves correspond to isoforms 1.1 and 1.2, respectively. Profiles are averaged over five independent replicas, and shaded regions indicate ±2 standard deviations. (B,C). Present density plots of the radius of gyration as a function of the Interdomain angle, with B. corresponding to isoform 1.1 and C. refer to isoform 1.2.

To obtain atomistic insights into the conformational energy landscape along the transition between the open and closed states, we projected the simulated ensembles on a two-dimensional coordinate space using ELViM. Figure 3 presents the ELViM projections for both isoforms. The phase of space is colored by interdomain distance, with red indicating open conformations and blue closed conformations. To ensure comparability between isoforms, the distance range was fixed between 20 and 70 Å. To highlight some of the frequently sampled conformations in the open, closed, and transition states, we employed the LCS tool. Consistent with the free-energy profiles discussed previously, isoform 1.2 exhibits more frequent transitions between the open and closed regions of the phase space. This behavior is reflected by a higher density in the transition region compared to isoform 1.1 (Figure 3B and D), indicating greater conformational accessibility and lower energetic cost for structural rearrangement. Taken together, the free-energy profiles (Figure 2) and the ELViM projections (Figure 3) provide a consistent and complementary description of the conformational landscapes of the two isoforms. While isoform 1.1 explores a more confined region of phase space, with a slight predominance of closed conformations, isoform 1.2 spans a broader region connecting open and closed conformations. It is noteworthy that the five amino acid sequence difference in the short helical segment, far away from the interdomain linker, reshapes the underlying energy landscape of CBD12 (Figure 1), biasing isoform 1.2 toward enhanced conformational flexibility.

**Figure 3:**
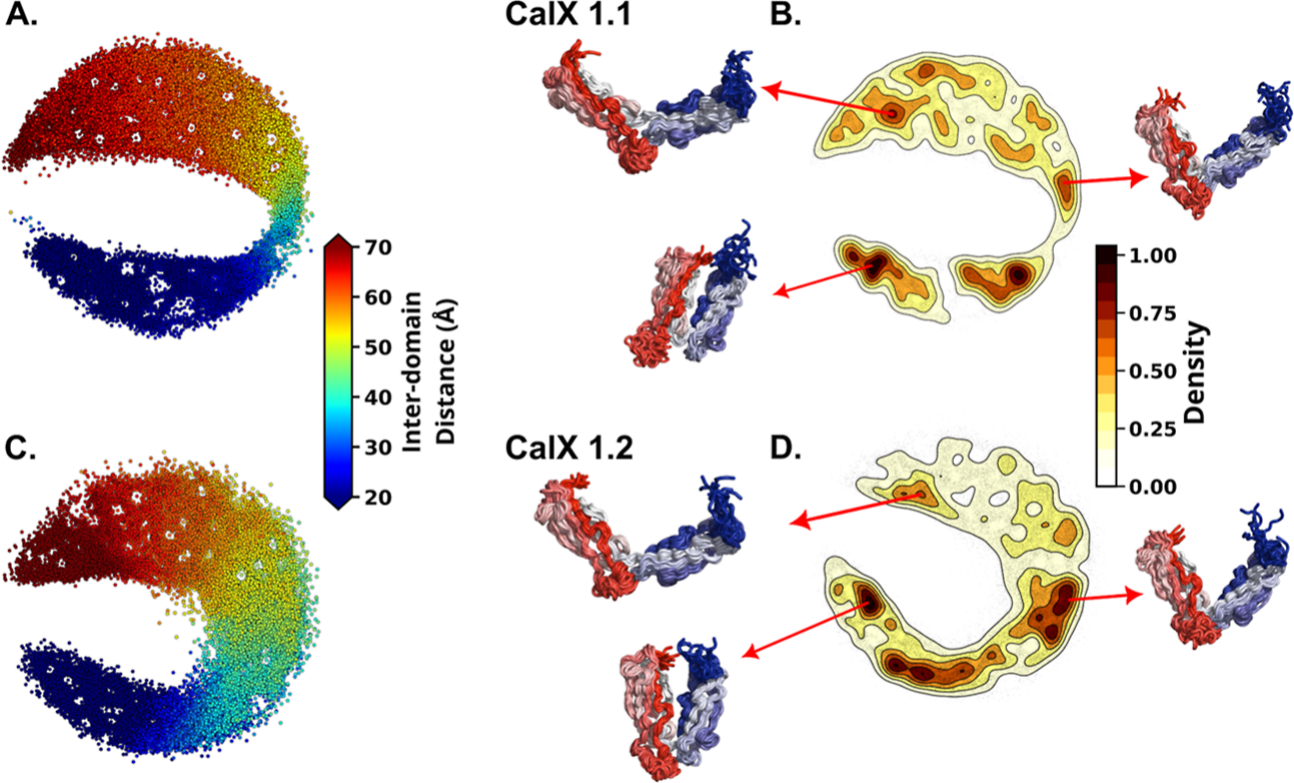
ELViM projecton to dual-basin SBM. Projections A and B correspond to isoform 1.1, whereas projections C and D correspond to isoform 1.2. In projections A and C, the ELViM map is colored according to the Interdomain Distance: colors near blue represent closed conformations, while colors near red indicate open conformations. In projections B and D, the point-density distribution is shown; darker colors (close to black) indicate regions of higher density, whereas lighter colors (toward white) represent regions of lower local density. These analyses were performed using an all-atom structure-based model without electrostatics (no charges and no ions).

To investigate how electrostatic screening and presence of a bound calcium ion modulate this intrinsic landscape, a Debye–Hückel (DH) potential was added to the dual-basin SBM, while calcium binding was modeled with a harmonic restrain to its crystallographic site. The impact of the DH term is shown in Figure 4A–C. The inclusion of long-range electrostatic screening alters the balance between the open and closed basins. As a consequence, the attractive well depths of both states were recalibrated to properly capture the transition. As described in the Methods section, the parameters ϵ_*ij*_^open^ and ϵ_*ij*_^closed^ associated with single-Gaussian contacts were adjusted so that the open and closed states occurred with comparable probability along the interdomain coordinate.

**Figure 4:**
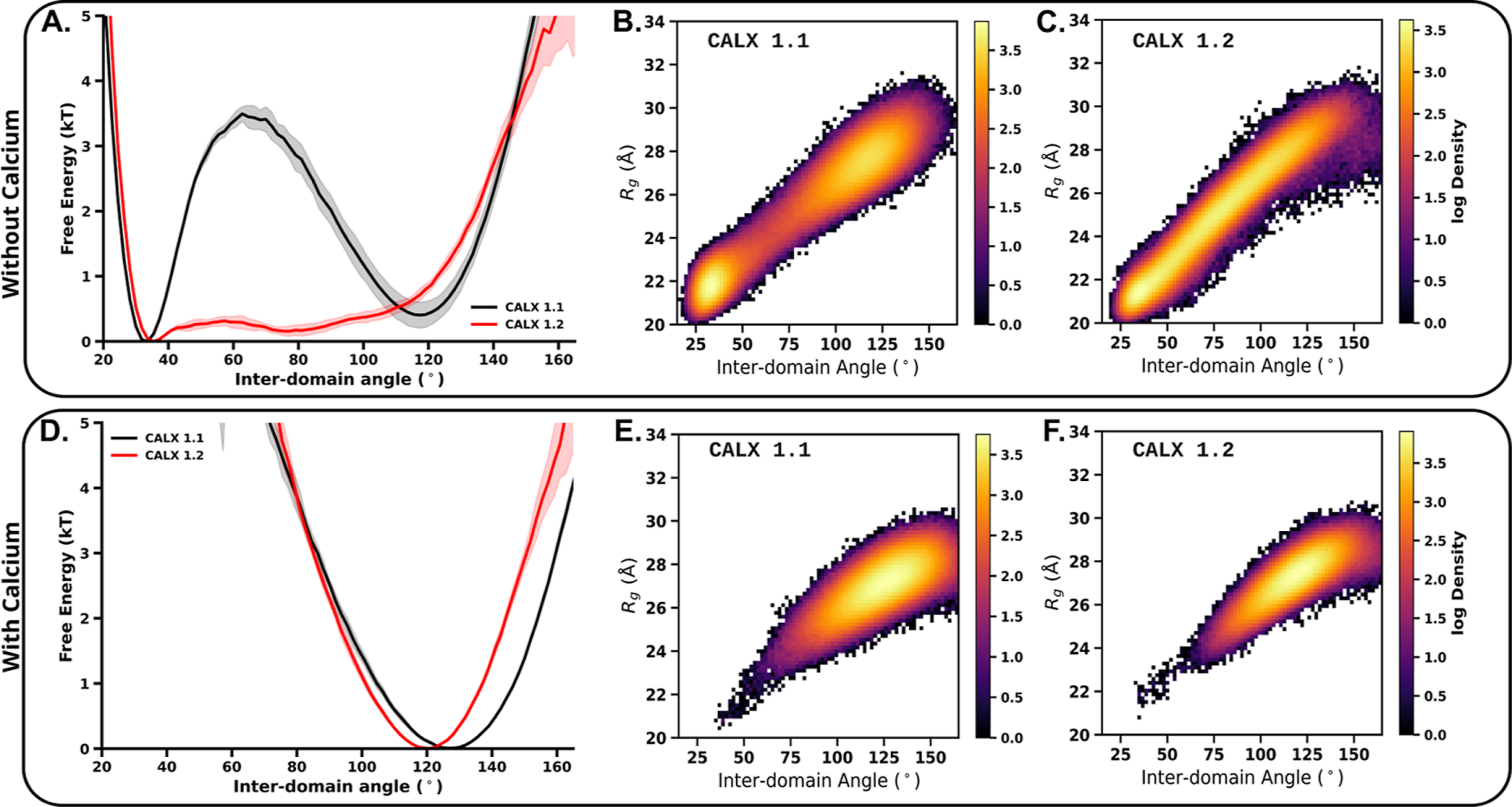
All-atom dual basin SBM with electrostatics potential. Panels A–C show the results obtained in the absence of calcium, while panels D–F include the effect of calcium. Black curves correspond to isoform 1.1, and red curves correspond to isoform 1.2. Panels A and D display the free energy as a function of the interdomain angle. Profiles are averaged over five independent replicas, and shaded regions indicate ±2 standard deviations. Panels B, C, E, and F show density plots of the radius of gyration as a function of the interdomain angle. Panels B and E correspond to isoform 1.1, whereas panels C and F correspond to isoform 1.2.

For isoform 1.1, the addition of the DH potential does not significantly change the free energy barrier compared to Figure 2A, indicating that electrostatic screening has a limited effect on the conformational equilibrium. In contrast, isoform 1.2 exhibits a clear reduction in the free-energy barrier upon inclusion of the DH term. This effect likely reflects how screened long-range interaction reshape the relative stabilization of intermediate conformations involving the two domain and the FG-loop, thereby enhancing the accessibility between the open and closed states.

Titration experiments monitored by solution NMR spectroscopy and calorimetry (ITC) reported by Cardoso et al.^16^ showed that calcium binding at the CBD1 domain is highly cooperative, supporting the assumption that the four calcium ions occupy the binding site simultaneously. Based on this high cooperativity, we modeled the fully bound state as described in the Methods section.

When the calcium binding sites in CBD1 are occupied, both CBD12 isoforms display a pronounced shift toward the V-shaped open conformation (Figure 4D–F). This behavior indicates that the presence of bound calcium ions stabilizes the open basin and restricts the conformational dynamics of the bound state, substantially reducing the probability of transitions toward the closed conformation.

Thus, although isoform 1.2 displays greater conformational flexibility in the absence of calcium and under screened electrostatic conditions, full calcium occupancy shifts the conformational equilibrium toward the active, V-shaped arrangement in both isoforms. This behavior is consistent with experimental observations that calcium binding stabilizes CBD12 in the open conformation.^25,26^ Additional simulations with weaker restraint strengths (*K* = 100 kcal mol^–1^ nm^–2^, *K* = 50 kcal mol^–1^ nm^–2^ and *K* = 10 kcal mol^–1^ nm^–2^) revealed only modest changes in the free-energy profiles (Supplementary Figure S3). Isoform 1.1 remained largely unaffected across the tested force constants, whereas isoform 1.2 exhibited increased conformational fluctuations at the weakest restraint strength, including a broader open basin and the emergence of a shallow minimum in the closed region. Nevertheless, the overall preference for open-like conformations was preserved in both isoforms, indicating that the calcium-induced shift in the conformational equilibrium is not solely a consequence of an overly restrictive harmonic constraint.

We used solution NMR spectroscopy to provide experimental evidence that CBD12 1.2 exhibits greater intrinsic flexibility than the 1.1 isoform. To this end, we compared the ^1^H–^15^N TROSY spectrum of the two isoforms. As shown in Figure 5, the NMR spectra of CBD12 1.1 and 1.2 are highly similar, indicating that the two proteins have the same overall structure. The change of 5 amino acids is expected to introduce peak shifts; however, in the absence of overall structural changes, such shifts were expected to be small. Examples of CBD12 1.1 cross peaks that could not be traced back in the 1.2 isoform NMR spectrum are indicated by green arrows. Peak picking indicated that CBD12 1.2 lacks 20 cross peaks compared to CBD12 1.1. The absence of peaks could be explained by conformational changes occurring on the microsecond to millisecond time scale, or by several low populated conformational species in slow exchange equilibrium with each other resulting in signals at the noise level. These observations are consistent with CBD12 1.2 exhibiting greater conformational heterogeneity as observed in the SBM simulations.

**Figure 5:**
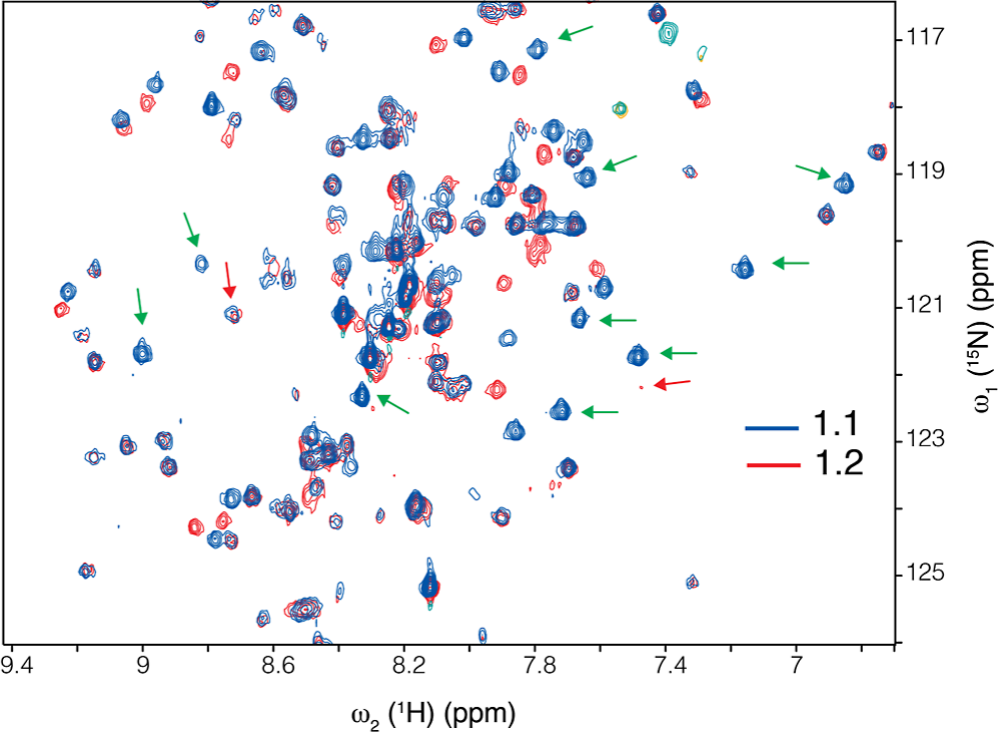
^15^N-TROSY NMR spectrum of the isoforms 1.1 (blue) and 1.2 (red) of CBD12 recorded at 800 MHz. Peaks colored in cyan (CBD12 1.1) or orange (CBD12 1.2) are negative due to folding effects. Examples of peaks that exist in the spectrum of the 1.1 isoform but could not be easily traced back in the spectrum of the 1.2 isoform are indicated by green arrows, while weak CBD12 1.2 peaks are indicated by red arrows.

## Conclusions

The dual-basin SBM simulations presented here provide atomistic insights into the interdomain motions exhibited by CBD12. In these simulations, the equilibrium between the open and closed CBD12 conformations was extensively sampled. The calculated free-energy landscapes show that in the free state CBD12 displays considerable conformational heterogeneity. Specifically, the 1.1 isoform exhibits two well-defined free-energy minima corresponding to the closed conformation and opened conformations, separated by a barrier of approximately 3.5 kT. This result is consistent with our previous interpretation of backbone ^1^H–^15^N RDCs, which indicated a predominance of closed interdomain conformations in the absence of calcium.^27^ In contrast, the 1.2 isoform samples a wider range of interdomain arrangements between the open and closed conformations, resulting in a smoother free-energy landscape and increased conformational flexibility. This behavior is consistent with the broader NMR resonances observed for isoform 1.2, which indicate enhanced conformational heterogeneity relative to isoform 1.1. These results, therefore, provide a structural interpretation for the increased line broadening observed in the NMR spectrum of isoform 1.2. Notably, the SBM simulations indicate that calcium binding to CBD1 stabilizes the V-shaped open conformation (TOC Figure). This behavior is fully consistent with experimental observations reported for NCX and CALX CBD12 constructs as well as for the full-length NCX1.4.^9,25,27,28,53^

## Supplementary Material



## Data Availability

All Trajectories and input files, as well as their descriptions, are publicly available at: 10.5281/zenodo.19322516. The ELViM method is available on GitHub (https://github.com/VLeiteGroup/ELViM). The following open-source software was used in this study: VMD^55^ and Pymol^56^ for structural visualization, and MDTraj^57^ for molecular dynamics trajectory processing.

## Notes

Additional free-energy profiles evaluate the sensitivity of the conformational landscape to variations in native–contact interaction strengths, convergence analyzes of the free-energy calculations, assessment of the effect of harmonic restraint strengths used to model calcium coordination, and detailed protocols for the all-atom molecular dynamics simulations employed to generate the reference structures for the structure-based model simulations (PDF)
