# Supplementary material for: Probing Charge-Controlled Inter-Domain Flexibility: Integrating Experimental and Coarse-Grained Approaches
Source: J Chem Inf Model. 2026 Jun 29;66(13):7651–9. doi: 10.1021/acs.jcim.6c00989 (PMC13370880; doi:10.1021/acs.jcim.6c00989)
Supplement: Supplementary file 1 [file ci6c00989_si_001.pdf]

Supporting Information: Probing Charge-Controlled Inter-Domain Flexibility: Integrating Experimental and Coarse-Grained Approaches

Larissa M.F. Adolfo,[†] Rafael G. Viegas,[‡] Mario A. R. Pineda,[¶,§] Delaram Taghavi,[§] Phelipe A. M. Vitale,[§] Roberto K. Salinas,[*,§] and Vitor B.P. Leite[*,‖]

†*São Paulo State University, São José do Rio Preto, Brazil*

‡*Federal Institute of Education, Science and Technology of São Paulo (IFSP), Catanduva, São Paulo, 15808-305, Brazil*

¶*Bio-science school, Universidad Nacional de Colombia, Medellin, Colombia*

§*Institute of Chemistry, University of São Paulo, São Paulo, Brazil*

‖*Institute of Chemistry, São Paulo State University, Araraquara, Brazil*

E-mail: rsalinas@usp.br; vitor.leite@unesp.br

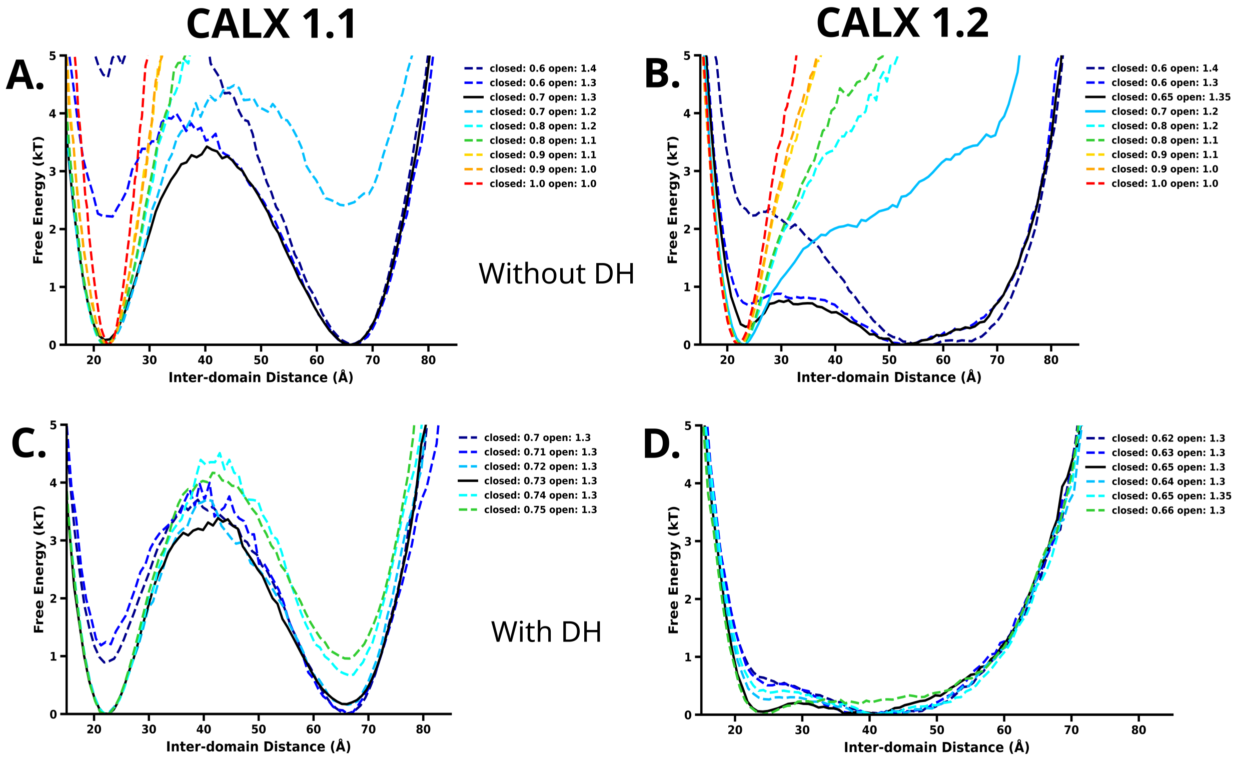

Figure S1: Sensitivity of the free-energy profiles to the scaling of the attractive well depths $\epsilon_{ij}^{\mathrm{open}}$ and $\epsilon_{ij}^{\mathrm{closed}}$. Uniform rescaling of the well-depths modifies the relative basin populations and conformational fluctuations while preserving the overall qualitative features of the conformational landscape. Panels A and C correspond to isoform 1.1 and panels B and D correspond to isoform 1.2. Panels A and B show the free-energy profiles obtained without eletrostatic potential and panels C and D correspond to simulations including the eletrostatic potential.

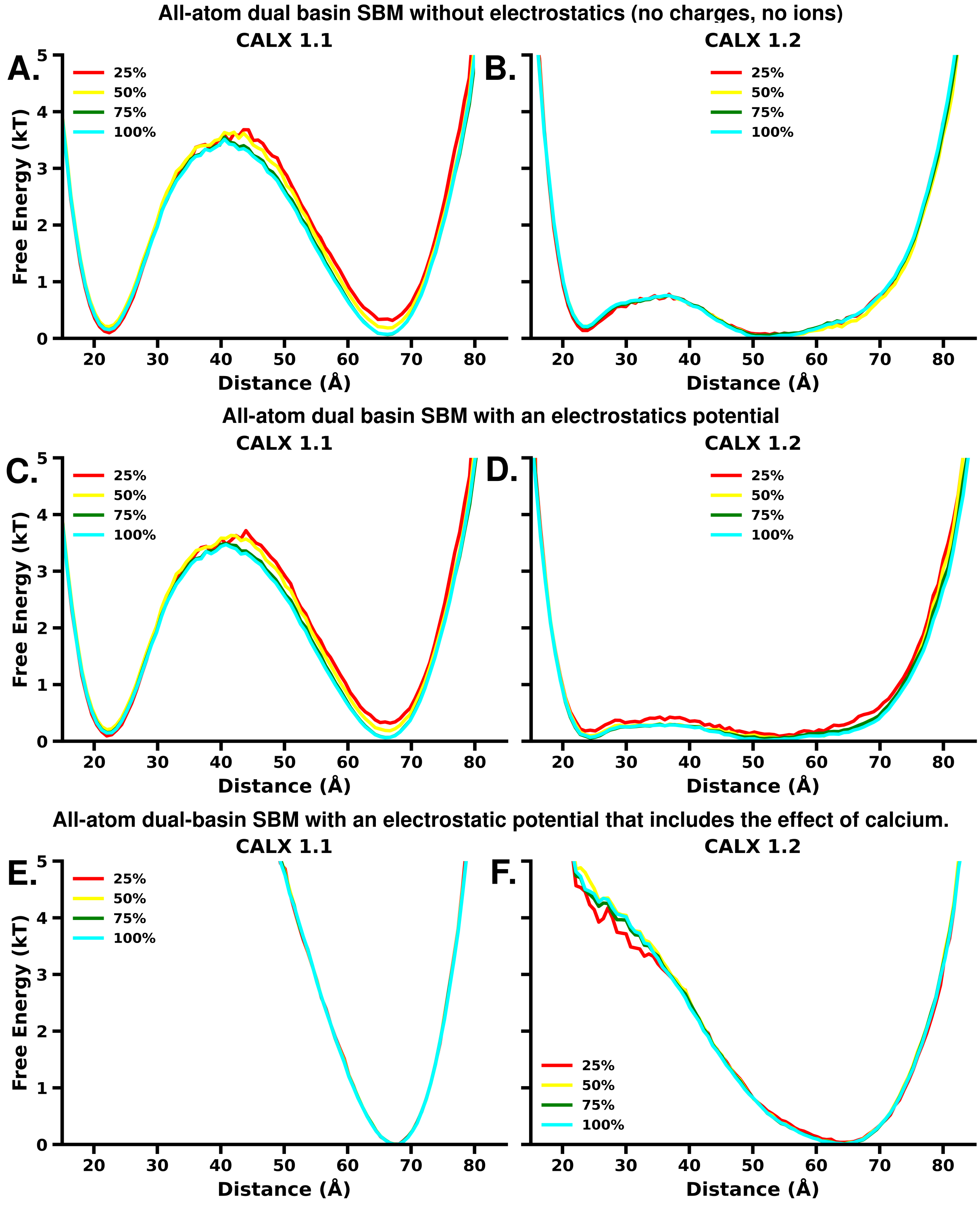

Figure S2: **Convergence of free-energy profiles along the inter-domain distance.** Free-energy profiles as a function of the inter-domain distance for isoform 1 (panels A, C, E) and isoform 2 (panels B, D, F). The profiles were computed using increasing fractions of the trajectories (25%, 50%, 75%, and 100%) to assess convergence. Each curve represents the average over five independent replicas (200,000 frames per replica). The overlap between profiles obtained at different trajectory lengths demonstrates that the free-energy landscapes are well converged.

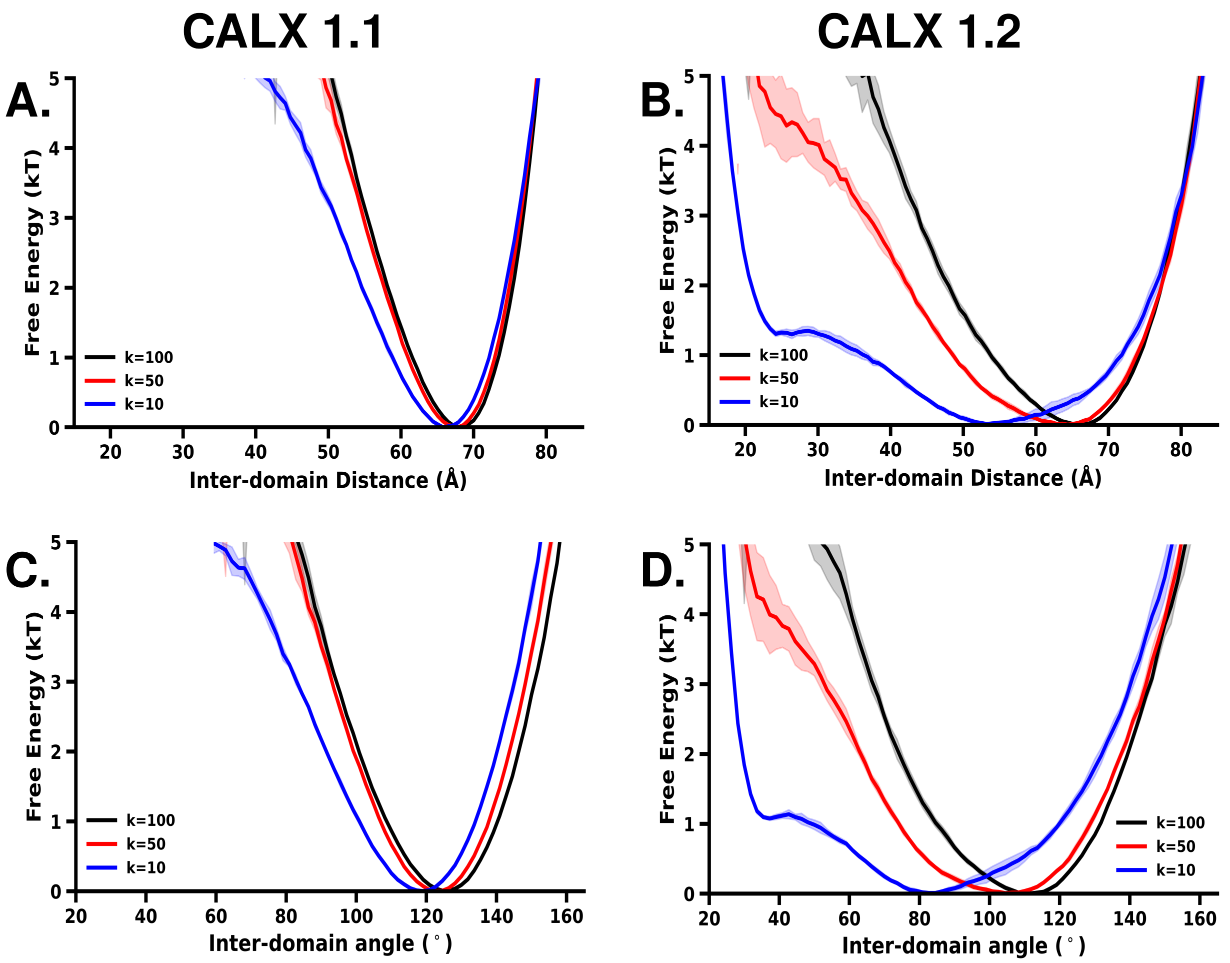

Figure S3: Effect of the harmonic restraint strength used to model calcium coordination. Free-energy profiles as a function of inter-domain distance (A,B) and angle (C,D), for isoforms 1.1 and 1.2 under different restraint strengths ($k = 100$, 50, and 10 $kcal \cdot mol^{-1} \cdot nm^{-2}$). Although weaker restraints increase conformational fluctuations, particularly in isoform 1.2, both systems remain strongly biased toward the open state, indicating that the effect is robust and not dependent on an excessively strong harmonic constraint.

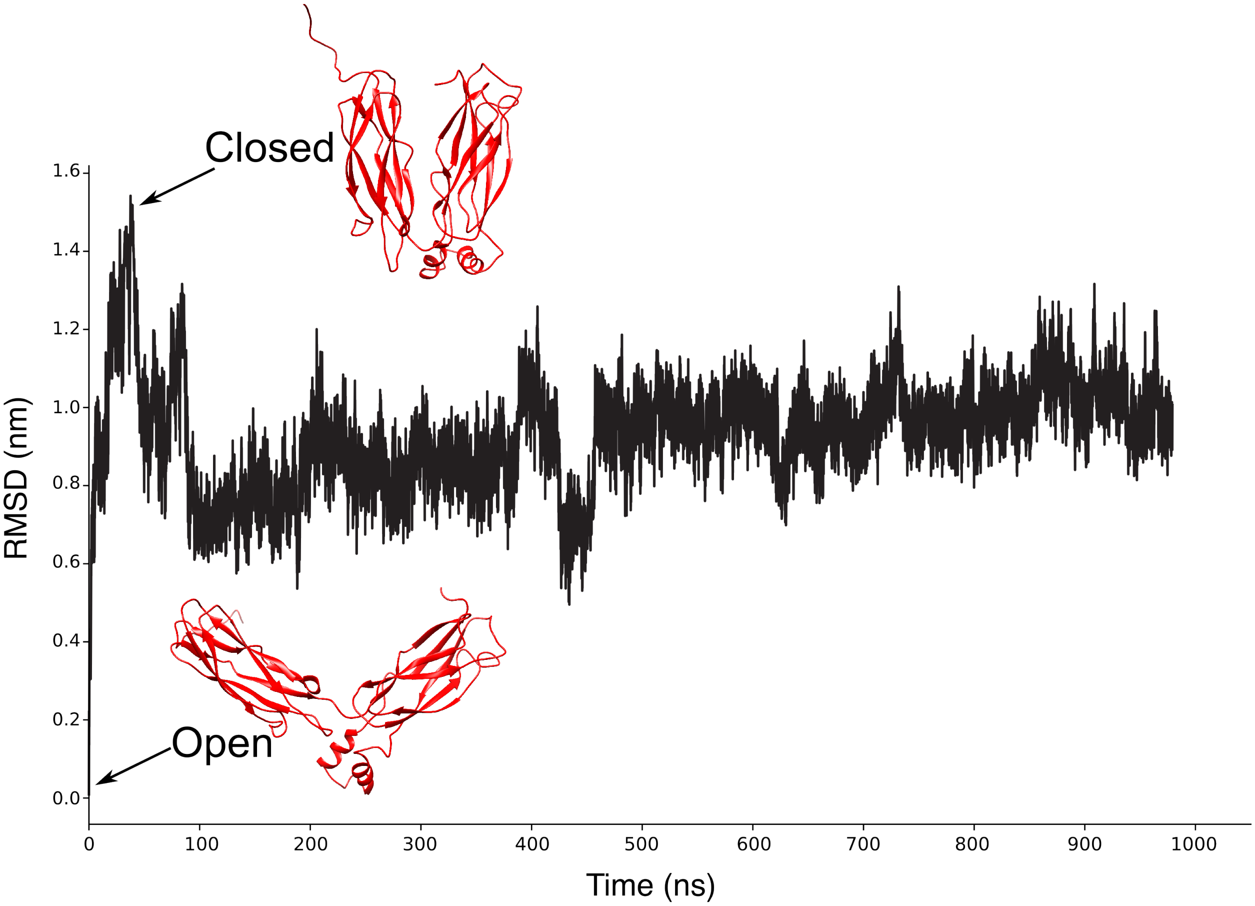

Figure S4: All-atom molecular dynamics simulation of CBD12 1.2. This simulation was carried out in explicit solvent using GROMACS 2018.6 and the AMBER99SBnmr1ILDN force field. The initial coordinates were retrieved from the PDB 3RB7 chain A, corresponding to the open conformation. Calcium ions were deleted. Missing loops were modelled with Robetta. The model was initially placed in a cubic box at 10Å from the edges and solvated with 46389 water molecules using the explicit water model TIP3P. The protein charges at pH 7.5 were determined with Propka 3.0. The system was neutralized with 0.2 M of NaCl. Long-range electrostatic and van der Waals interactions were cut off at 1.0 nm using the PME algorithm with 1.6 Å spacing. This system was simulated at 298 K for 1 ns at constant temperature conditions (NVT), followed by 1 ns under constant pressure conditions (NPT) and then allowed to evolve for 1.0 μs at 298 K. The snapshot at 40 ns was taken as initial model for the closed conformation for the structure-based model simulations, indicated by the arrow.
